# Application Progress of Electron Beam Radiation in Adsorption Functional Materials Preparation

**DOI:** 10.3390/molecules30051084

**Published:** 2025-02-27

**Authors:** Jie Gao, Xiang Li, Tao Chen, Yuan Zhao, Houhua Xiong, Xiaobing Han

**Affiliations:** Hubei Key Laboratory of Radiation Chemistry and Functional Materials, School of Nuclear Technology and Chemistry & Biology, Hubei University of Science and Technology, Xianning 437100, China; gaojie2019@hbust.edu.cn (J.G.); danny_lxx@163.com (X.L.); taochen518@163.com (T.C.); zhyf308@hbust.edu.cn (Y.Z.)

**Keywords:** electron beam radiation, adsorption functional materials, preparation, application

## Abstract

To solve the problems of water and air pollution, adsorption functional materials (ASFMs) have been extensively investigated and applied. Among the preparation methods of ASFM, electron beam radiation (EBR) has attracted much attention for its high efficiency, environmental friendliness, and wide applicability. Based on the introduction of the application of EBR technology, the EBR preparation of ASFM is summarized by grafting and cross-linking. Secondly, the application of corresponding ASFM for the adsorption of metal ions, inorganic anions, dyes, drugs and chemical raw materials, and carbon dioxide is summarized systematically. Then, the adsorption mechanisms of ASFM are illustrated, according to the different pollutants. Finally, the progress, issues, and prospects of EBR technology for ASFM preparation are discussed.

## 1. Introduction

Environmental pollution, especially water pollution and air pollution, has become a serious threat to human health [1,2,3]. Water pollutants mainly include heavy metals, inorganic anions, organic dyes, drugs, and other chemical materials [4,5,6,7]. The harmful substances in the air mainly contain carbon dioxide, nitrogen oxides, sulfurides, and volatile organic compounds [8,9,10,11]. To address the environmental pollution caused by these harmful substances, technologies such as precipitation, adsorption, chemical oxidation, biological oxidation, advanced oxidation, radiolysis, and reverse osmosis have been widely studied and applied [12,13,14,15,16,17]. Among these technologies, the adsorption methods have received extensive attention due to their advantages of simple operation, high efficiency, and low cost.

Functional materials used for pollutant adsorption mainly include natural adsorbents, synthetic adsorbents, and grafted adsorbents [18,19,20]. Natural adsorbents refer to materials from nature that possess adsorption properties, such as minerals of diatomite, sepiolite, pumice, and biomass of cellulose, sodium alginate, chitosan, lignin, tannin [21,22,23,24,25,26,27,28]. Though the cost of natural adsorbents is relatively low, their adsorption capacity is always low. The most popular adsorbents are synthetic adsorbents, including inorganic zeolite, graphene, graphene oxide, biochar, organic ion exchange resins, chelating resins, hydrogels, metal–organic frameworks, and covalent organic frameworks [29,30,31,32,33,34]. These synthetic adsorbents have excellent adsorption performance due to their large surface area and rich functional groups; however, the corresponding raw materials are always expensive and the synthesis process is relatively complex. The grafted adsorbents have attracted much attention due to their simple synthesis process and good adsorption properties [20,35,36]. The grafted adsorbents are always obtained via the functionalization of a low-cost inorganic/organic matrix with functional molecules or functional polymers.

To achieve good adsorption behavior toward different pollutants, synthetic adsorbents and grafted adsorbents have been developed rapidly. However, both the synthetic adsorbents and grafted adsorbents have been synthesized through conventional chemical methods at this stage [33,34,35,36]. There are many problems with the traditional chemical methods of adsorbent preparation, the most serious of which is the use of large amounts of toxic solvents that cause serious environmental pollution. In addition, there are problems such as long reaction times and low yields [29,30,31,32,33,34]. For the grafted adsorbents, the range of applicable substrates for traditional chemical grafting methods is limited. Therefore, in recent years, various clean radiation technologies have been extensively studied in the preparation of adsorption functional materials (ASFMs), including microwave, ultraviolet, γ-rays (^60^Co), and electron beam (EB) [37,38,39,40,41,42]. Microwave and ultraviolet radiation exhibit low-energy/poor penetrability, and ^60^Co radiation shows high-radioactivity and poor controllability, making electron beam radiation (EBR) a promising method for the preparation of ASFM [20,40,42].

Recently, significant progress has been made in the preparation of ASFM using EBR technology, because it is environmentally friendly, highly efficient, and widely applicable [20,40,42]. Though many works about the ASFM development and EBR application have been published, there are rarely reviews about the EBR preparation and application of ASFM. Now is an appropriate time to summarize the progress of corresponding theories, applications, problems, and developments.

## 2. Application of Electron Beam Radiation (EBR) Technology

**Figure 1:**
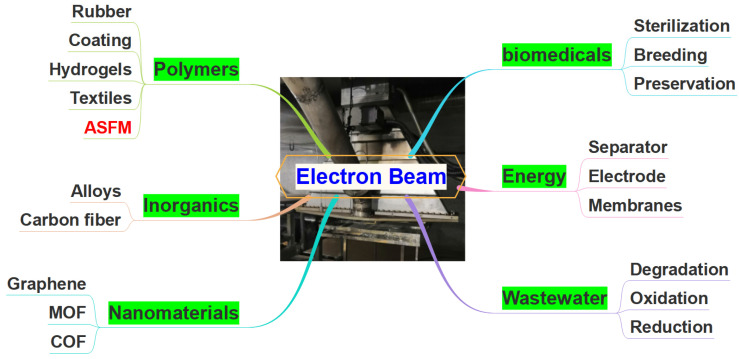
Application of EBR technology.

EBR is a technology that utilizes the high-energy electron beam generated by an electron accelerator to interact with matter or an organism, thereby achieving changes in structure and properties [43]. The EBR technology does not use any chemical substances or additives, thus it will not cause pollution or harm to the environment. In addition, the EBR possesses good penetrability and relatively short processing time. Due to its environmentally friendliness and high efficiency, EBR technology has been extensively used in the fields of different materials preparation, biomedicals, energy, and wastewater treatment (Figure 1).

Many materials can be prepared with EBR technology, such as polymeric materials, inorganic materials, and nanomaterials. For the polymeric materials preparation, the EB can conduct the curing of rubber, coating, and hydrogels [44,45], as well as the surface modification of textiles [46]. EBR can also be used for the preparation of ASFM through the polymerization of a functional monomer, or the grafting of a functional monomer onto different matrices [20,40]. In addition, the alloys melting in additive manufacturing [47] and carbon fibers originating from polyacrylonitrile fibers [48] can as well be obtained with EBR technology. Recently, novel nanomaterials such as graphene [49], metal–organic frameworks (MOFs) [50], and covalent organic frameworks [51] have been fabricated with EBR technology.

In the biomedical field, EBR technology has been widely used for the sterilization of medicinal materials and sanitary supplies, preservation of food, and crop breeding [52,53]. For the development of the energy industry, EBR technology was applied for the modification of an electrode/separator (lithium-ion batteries) [54] and anion exchange membranes (fuel cells) [55]. Now, efficient EBR technology has been used for wastewater treatment through chemical reactions, including the degradation of dyes, oxidation of organic compounds, and reduction in metal ions [56,57].

## 3. Adsorption Functional Materials (ASFMs) Prepared with EBR Technology

**Figure 2:**
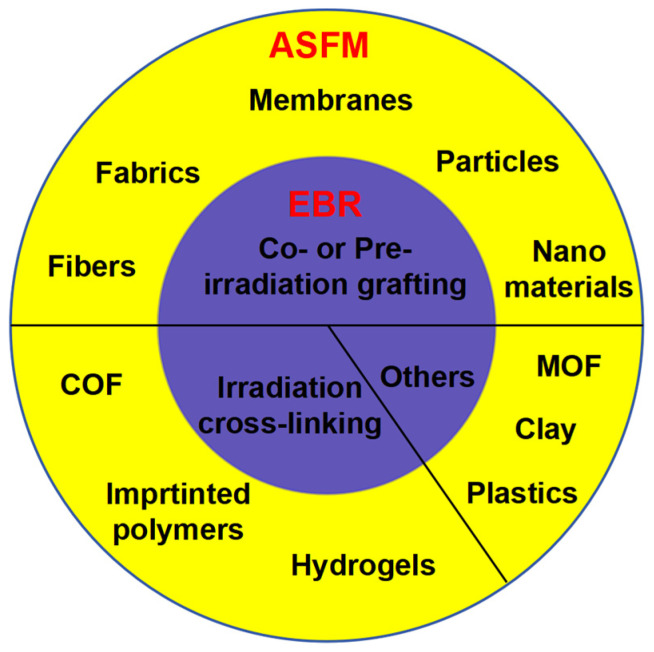
ASFM prepared with EBR technology.

According to the difference in the EBR mechanism, the ASFMs synthesized with EBR can be divided into three groups (Figure 2). The major group of ASFM is obtained through co/pre-irradiation grafting, and the main matrix includes fibers, fabrics, membranes, particles, and nanomaterials [20,40]. The second group of ASFM is fabricated via irradiation cross-linking, such as hydrogels [58], imprinted polymers [59], and COF [51]. The last group is fabricated through the intrinsic structure transformation, including an MOF [50], bentonite [60], and microplastics [61].

### 3.1. ASFM Prepared with EBR Grafting

**Figure 3:**
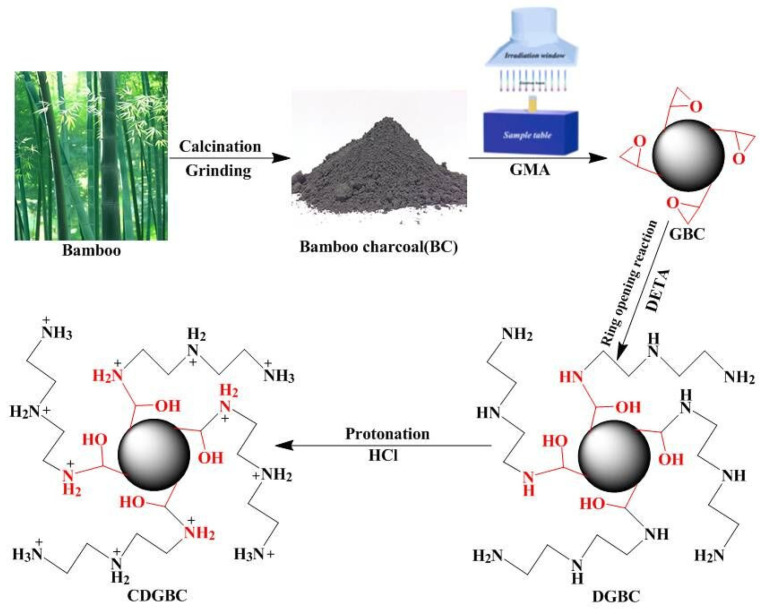
Biochar-based Cr_2_O_7_^2−^ adsorbents synthesized through co-irradiation grafting [62].

The EBR-induced grafting includes co-irradiation grafting and pre-irradiation grafting [20,40]. For the co-irradiation grafting, the organic or inorganic matrix is immersed into the solution of functional monomer, and it is irradiated simultaneously. Both the matrix and the monomer can form active radicals, and they can initiate grafting reactions and homopolymerization, respectively. The process of co-irradiation grafting is relatively simple, but the homopolymerization of functional monomers can reduce the grafting efficiency. In our previous work, protonated amino-bamboo char was fabricated via EBR grafting for the removal of Cr_2_O_7_^2−^ (Figure 3) [62]: glyceride methacrylate (GMA) was grafted onto bamboo char through co-irradiation firstly, then amino groups were introduced via the reaction of epoxide group and diethyltriamine, and finally the adsorbents were synthesized with the protonated of amino functional groups. For the aminated bamboo char, the grafted epoxide groups (C-O vibration at 1079 cm^−1^) changed to C-N vibration (1064 cm^−1^). After the protonation of amine, the peak intensity of the nitrogen-containing group decreased obviously. Due to the strong electrostatic attraction between the obtained adsorbents and dichromate anion, the maximum adsorption capacity reached 169.13 mg/g for Cr_2_O_7_^2−^.

**Figure 4:**
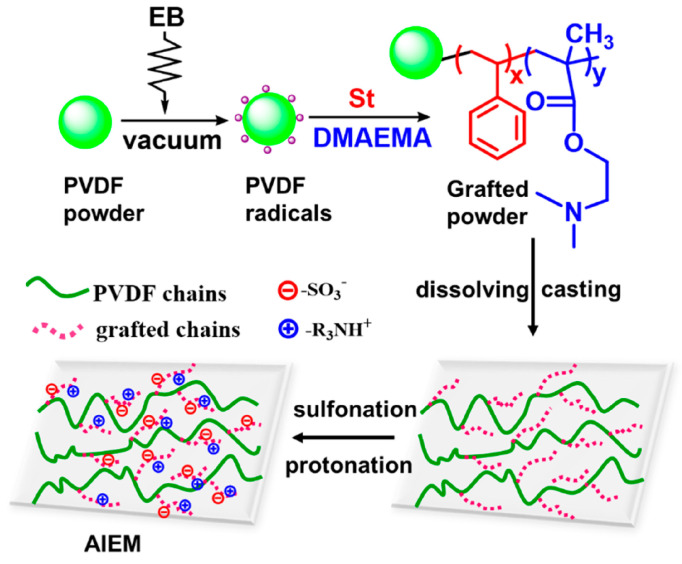
PVDF-based VO^2+^ exchange membranes are obtained with pre-irradiation grafting [63].

For the pre-irradiation grafting, the matrix was irradiated under inert media to generate active radicals and then immersed into the solution of functional monomer to initiate a grafting reaction. The homopolymerization of the functional monomer is inhibited in pre-irradiation grafting, but the process is relatively complex [20,40]. Amphiphilic poly(vinylidene difluoride) (PVDF)-based membranes with ion exchange properties were fabricated via pre-irradiation grafting for a vanadium battery (Figure 4) [63], and the PDVF matrix was pre-irradiated to form active radicals firstly and then a functional monomer (dimethylaminoethyl methacrylate, DMAEMA) was added to graft ion exchange groups. The highest grafting yield was 33.6%, and the ion exchange capacity of the synthesized amphoteric ion exchange membranes (AIEM) was 1.43 mmol/g.

### 3.2. ASFM Prepared with EBR Cross-Linking

**Figure 5:**
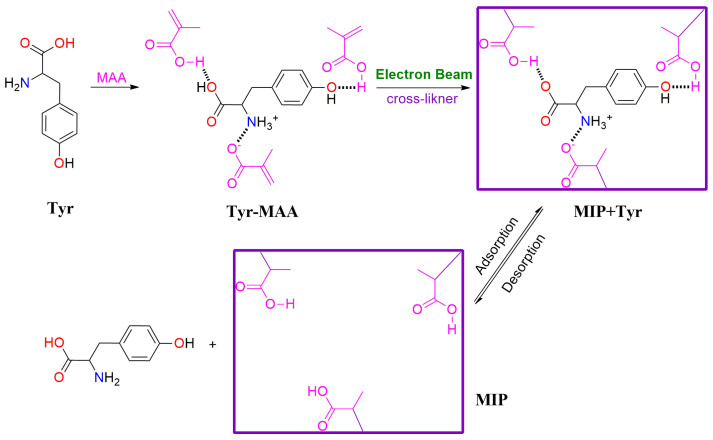
Tyrosine-imprinted polymers fabricated with EBR-induced cross-linking [65].

The EBR technology-induced cross-linking includes the formation of functional hydrogels, imprinted polymers, and COF [51,58,59]. For functional hydrogels, the cross-linked network formed between polymer chains is initiated by an electron beam [20]. For imprinted polymers and COF, the cross-linked network formed between monomers with one and multiple functional groups [40]. Trimethylolpropane trimethacrylate cross-linked acrylamide and acrylic acid copolymers were fabricated using EBR [64]; the synthesized hydrogel possesses a swelling ratio as high as 8500%, and exhibits a relatively high adsorption capacity toward Cu^2+^ (66 mg/g) and Cr_2_O_7_^2−^ (128 mg/g). In our previous work, tyrosine imprinted polymers were prepared with EBR-induced cross-linking (Figure 5) [65]; the non-molecularly imprinted polymers exhibited a dense and smooth surface, but the tyrosine-imprinted polymers showed a rough surface and porous structure. The highest adsorption capacity of 10.96 mg/g was reached with a dosage of 340 kGy. In addition, a high imprinting factor of 5.1 was obtained, as well as relatively high selective coefficients toward tryptophan (3.9) and phenylalanine (3.5). Crystalline porous COF was synthesized via EBR cross-linking under ambient conditions, with the functional monomers of 2,4,6-tris(4-formylphenoxy)-1,3,5-triazine and 1,3,5-tris(4-amino phenyl)-triazene [51], and the surface area of the synthesized COF as high as 738 m^2^/g.

### 3.3. ASFM Prepared with EBR-Induced Structure Transformation

The EBR technology can also induce intrinsic structure transformation of different materials, which will benefit the improvement of adsorption performance. ZIF-L crystals are promising adsorption functional materials; however, they are sensitive to water. Amorphous ZIF-L was obtained after exposure to EBR [50], and amorphized ZIF-L has excellent stability toward water. With the assistance of EBR, strong molecular repulsion was synthesized for bentonite clay, leading to an even dispersion and high adsorption toward Cr_2_O_7_^2−^ (324.3 mg/g) [60]. To address the pollution caused by waste microplastic, degraded polypropylene microplastics with functional groups were obtained through EBR technology [61]. The XRD peaks of the degraded polypropylene remained unchanged, while the intensity decreased obviously, which means the crystallinity of degraded polypropylene decreased significantly. In addition, the XPS results revealed the oxygen/carbon ratio reached 0.071 after irradiation, which made the functionalized microplastics exhibit a high removal efficiency toward Pb^2+^.

## 4. Application of ASFM Prepared with EBR Technology

Due to its environmentally friendly and high efficiency, EBR has been extensively used in the preparation of ASFM [20,40]. In addition, the EBR technology can be conducted with different methods, including co/pre-irradiation grafting, irradiation cross-linking, and structure transformation. According to the difference in pollutants, the recent application progress of ASFM synthesized EBR technology for the removal of metal ions, inorganic anions, dyes, drugs, chemical raw materials, and others are summarized.

### 4.1. Adsorption of Metal Ions

According to the properties of metal elements, the metal ions pollutants can be divided into heavy metal ions, precious metal elements, rare earth ions, and radioactive metal ions. The major factor for the designing of ASFM is the charge of metals. Most metal ions possess a positive charge, and only a few metal-containing ions exhibit a negative charge, such as Cr(SO_4_)^2−^, Cr_2_O_7_^2−^, AuCl_4_^−^, and radioactive metal anions [5,13,18].

#### 4.1.1. Metal Cations Adsorption

The progress of metal cations adsorption with ASFM fabricated via EBR technology is summarized in Table 1, the metal cations include heavy metal, precious metal, and rare earth. The major EBR technology is pre-irradiation and co-irradiation, and the main matrix is based on natural and synthetic polymers.

EB-irradiated sheep wool was fabricated for the adsorption of Co^2+^ [66]; except for the existing amino and hydroxyl functional groups, additional salts were formed in the irradiated wool, which are favorable for the adsorption of Co^2+^. In addition, ten isotherm models were fitted for the adsorption process, and the highest adsorption capacity of 29.96 mg/g was obtained at 153 kGy. Porous diatomite was chosen as a matrix for the preparation of Cu^2+^ absorbent with EBR grafting [67], and the synthesized thiacalix [4]arene @polyacrylic-g-diatomite (TC4A/PAA/diatomite) exhibited a maximum capacity of 123.3 mg/g at 90 kGy. In our previous work, iminodiacetic acid functionalized ramie was fabricated via co-irradiation grafting for the removal of Pb^2+^ [68]; due to the strong interaction between Pb^2+^ and the functionalized ramie, a high capacity of 225.71 mg/g was obtained at 200 kGy. In our other work, an iminodiacetic acid grafted loofah sponge was prepared through co-irradiation for the adsorption of Cd^2+^ [69], and the synthesized absorbents exhibited an adsorption capacity of 169.81 mg/g with a dosage of 240 kGy.

L-cysteine and pentaethylenehexamie functionalized cellulose microsphere (CysMC and PMC) fabricated via pre-irradiation grafting have been reported by the group of Du [70]: the obtained absorbents exhibit good adsorption performance toward Hg^2+^. The maximum capacities are 264.55 and 169.49 mg/g for CysMC and PMC, respectively, and the adsorption kinetic follows the pseudo-second-order model. In another work, tannic acid functionalized cellulose was prepared for the recovery of Ga^3+^ and In^3+^ [71]. The adsorption capacity is relatively low (26.55 and 35.63 mg/g), but the selectivity is very high. Due to the stable chelating interaction, the fabricated absorbent can efficiently separate Ga^3+^ and In^3+^ from the mixture solution with Cu^2+^, Zn^2+^, and Ni^2+^.

L-cysteine irradiated grafted cellulose microsphere (CysMC) has also been used for the selective adsorption of Ag^+^ [72]. The prepared CysMC exhibits an adsorption capacity of 66.67 mg/g toward Ag^+^. In addition, the CysMC shows excellent selectivity of Ag^+^ against Mg^2+^, Ca^2+^, Ni^2+^, and Zn^2+^. 2-Aminomethyl-pyridine grafted cellulose (2-AMP) has been developed for the selective recovery of Ag^+^ from high saline solution [73], and the maximum adsorption capacity is 110.5 mg/g.

**Figure 6:**
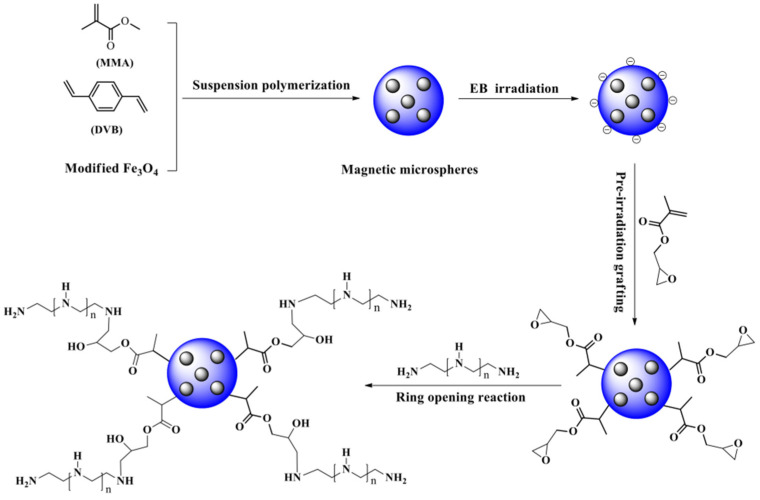
Magnetic PMMA-based Ce^3+^ absorbents prepared through co-irradiation grafting [75].

EBR-grafted synthetic polymeric microspheres for the adsorption of rare earth ions have been reported by our group [74,75]. Amino-phosphonic acid modified polystyrene microspheres (PS-PGMA-DETA) were fabricated for the removal of La^3+^ [74]: due to the strong chelating interaction between amino-phosphonic acid ligand and La^3+^, a high adsorption capacity of 288.69 mg/g was synthesized with the irradiation dosage of 240 kGy. In addition, aminated magnetic poly(methyl methacrylate) microspheres (PMMA-PGMA-PEI) were prepared for the adsorption of Ce^3+^ [75] (Figure 6). The nitrogen-containing groups as well exhibit strong chelating interaction toward Ce^3+^, and the highest adsorption capacity is 189.81 mg/g.

#### 4.1.2. Metal-Containing Anions Adsorption

The heavy metal chromium, precious metal aurum, and radioactive metal rhenium always exist as anionic complexes in aqueous solution. Recent progress of metal-containing anions adsorption with ASFM fabricated through EBR technology is summarized in Table 2.

EBR-degraded polypropylene microplastic was used for the adsorption of Cr_2_O_7_^2−^ [76]: due to the introduction of oxygen-containing groups, electrostatic and chelation interaction is formed between the degraded microplastic and Cr_2_O_7_^2−^. Based on the electrostatic and chelation interaction, tertiary amine and phosphate bifunctional groups modified polyethylene/polypropylene fibrous adsorbents were developed for the removal of Cr_2_O_7_^2−^ [77], with the maximum adsorption capacity of 38.43 mg/g and an adsorption efficiency of 92.7%. Glycidylmethacrylate and N-methyl glucamine co-grafted TiO_2_ were fabricated for the adsorption–photocatalytic reduction of Cr_2_O_7_^2−^ [78]: the highest removal rate (8.18%) was obtained within an hour at [Cr(VI)] = 10 ppm. EB-irradiated sheep wool was also used for the adsorption of Cr(SO_4_)_2_^−^ [79], and the chemical sorption of Cr(III) increased with the increase in irradiation dosage.

**Figure 7:**
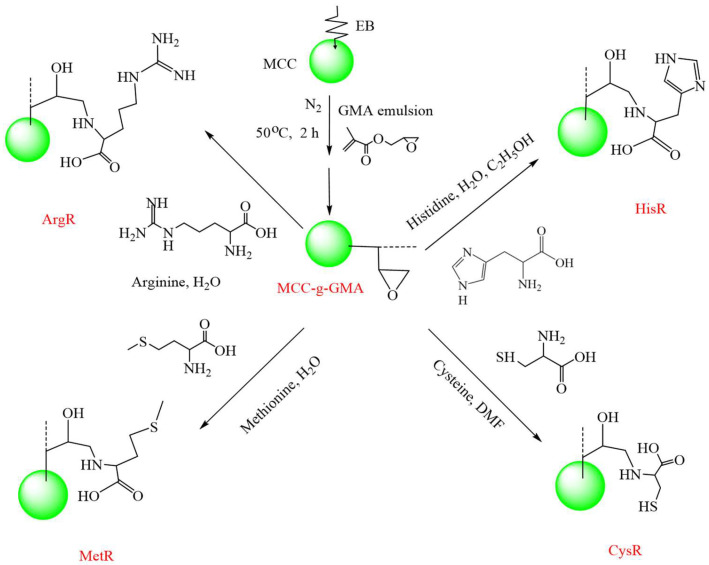
Cellulose-based AuCl_4_^−^ absorbents fabricated through pre-irradiation grafting [82].

Different EBR-grafted cellulose microspheres for the recovery of AuCl_4_^−^ have been reported by the group of Zhao [80,81,82]. L-cysteine modified cellulose microsphere (MCC-g-GMA-Cys) has been used for the efficient recovery of AuCl_4_^−^ [80], and the highest adsorption capacity reached is 714.28 mg/g. In addition, the synthesized adsorbents exhibit good selectivity of AuCl_4_^−^ against Ni^2+^ and Zn^2+^. Aminomethyl pyridine functionalized cellulose microsphere (n-AMPRs) have been fabricated for the recovery of AuCl_4_^−^ [81]; the adsorption capacity is in the order of *ortho*-AMPR > *meta*-AMPR > *para*-AMPR, which can be ascribed to the different chelation interaction. Different amino acid grafted cellulose microspheres (MCC-g-GMA-Amino) were developed for the selective recovery of AuCl_4_^−^ [82] (Figure 7), and the maximum capacity of ArgR, MerR, CysR, and HisR are 396.83, 549.45, 636.94, and 769.23 mg/g, respectively. In addition, the AuCl_4_^−^ in the leaching solution and gold slag waste can also be selectively recovered with these amino acid-modified cellulose microspheres.

Due to the huge environmental hazard, strategy for the recovery of radioactive metal waste is very important. Different EBR-grated cellulose microspheres were also developed for the recovery of radioactive metal ions [83,84,85,86]. Nucleobases such as xanthine, hypoxanthine, guanine, and adenine modified cellulose microspheres were fabricated for the removal of ReO_4_^−^ [83]: due to the strong electrostatic interaction, the obtained adsorbents had a capacity in the range of 23.43~194.0 mg/g. Amino guanidine functionalized cellulose (AGfC) had been prepared for the recovery of ReO_4_^−^ [84]; the maximum adsorption capacity of 264.55 mg/g was obtained at pH = 2.37. In addition, the obtained AGfC adsorbent can selectively separate trace ReO_4_^−^ from the leaching solution of uranium ore. A series of ionic liquids modified cellulose microspheres (MCC-[C_n_VIm]Br) were fabricated for the removal of ReO_4_^−^ [85], which exhibited a high recovery efficiency over a wide pH range. The highest adsorption capacity was 230.41 mg/g, and the recovery efficiency changed slightly after five times adsorption and resorption. Efficient and selective adsorption of ReO_4_^−^ was realized with gallic acid functionalized cellulose microspheres (MCGA) [86]; the maximum adsorption capacity of ReO_4_^−^ was 134.05 mg/g, and the separation factor of ReO_4_^−^/Mo_7_O_24_^6−^ was in the range 5.23~735.53.

### 4.2. Adsorption of Inorganic Anions

The excessive inorganic anions in water can not only cause ecological damage, but also pose a threat to human health. The inorganic anions removal with ASFM fabricated with EBR technology is summarized in Table 3.

The accumulation of fluorine in the human body will cause damage to the bone cells, odontoblasts, hard tissues, and osteoclasts, thus the removal of F^−^ in wastewater is an urgent problem. Carboxymethyl cellulose/multiwall carbon nanotube-loaded zirconium oxychloride gels (CMC + MWCCNT-ZrOCl_2_) were fabricated for the removal of F^−^ [87], the maximum adsorption capacity was 36.66 mg/g. In addition, the synthesized gels exhibit excellent selectivity, and the selectivity coefficients of F^−^ against NO_3_^−^, PO_4_^3−^, and SO_4_^2−^ are 140.34, 10.95, and 72.57, respectively. N-methylglucamine functionalized PE-PP fabric was developed for the adsorption of BO_3_^3−^ [88]; due to the strong hydrogen bonding formed between BO_3_^3−^ and the N-methylglucamine, the highest adsorption capacity was obtained at 23.78 mg/g. Chlorine disinfection can kill pathogenic microorganisms in water, but it forms harmful trichloroacetic acid. In the group of Li, four nano-oxide modified microcrystalline celluloses were prepared for the removal of Cl_3_CCOO^−^ [89], and the SiO_2_@MCC had a higher adsorption rate (83.27%) than the other three adsorbents. Compared with traditional disinfectants, chlorine dioxide has significant advantages in drinking water disinfection and deodorization, but it also forms chlorite, which is harmful to the human liver. This group has developed functionalized non-woven fabric for the adsorption of ClO_2_^−^ [90]; the removal efficiency is 89.00%, which is expected when used in the purification of drinking water.

**Figure 8:**
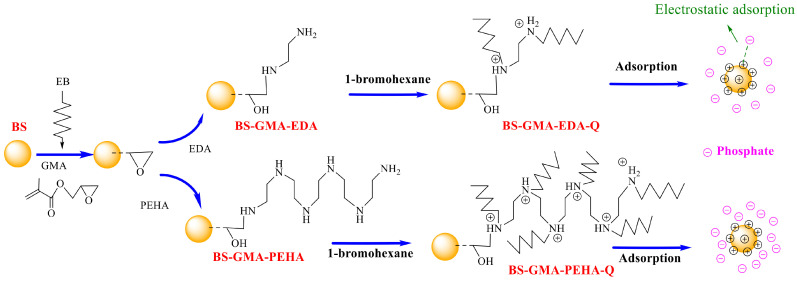
Bamboo-based PO_4_^3−^ absorbents fabricated through co-irradiation grafting [95].

As the phosphate (PO_4_^3−^) can promote the rapid growth of plants in water, thus excessive phosphate causes water eutrophication. Now, many adsorbents have been developed for the removal of PO_4_^3−^, including the ASFM fabricated with EBR technology. Acrylamide/ethylenediamine/phthalic anhydride grafted microcrystalline cellulose with loading Fe^3+^ was prepared for the adsorption of PO_4_^3−^ [91], and due to the electrostatic interaction between Fe^3+^ and PO_4_^3−^, the adsorption rate was as high as 89.7% under alkaline conditions. Different quaternary ammonium salt type adsorbents were fabricated for the removal of PO_4_^3−^ and have been reported by the group of Du [92,93,94,95]. Dimethylaminoethyl methacrylate directly grafted and quaterizated cotton linter (QCL) was prepared for the removal of PO_4_^3−^ [92]; the grafting yield was 69%, leading to a low adsorption capacity (36.13 mg/g) of PO_4_^3−^. Pentaethylene hexamine functionalized cotton linter was obtained with GMA-g-CL, and due to the high grafting yield of GMA (255%), the maximum adsorption capacity of the synthesized adsorbent reached 152.44 mg/g [93]. A similar phenomenon was observed for the quaternary ammonium salt adsorbent of the bamboo matrix. Methacrylic ethyltrimethyl ammonium chloride directly grafted bamboo fiber exhibited a low adsorption capacity of 67.48 mg/g toward PO_4_^3−^ [94], but the pentaethylene hexamine modified bamboo sawdust with grafted GMA possessed a very high adsorption capacity of 152.21 mg/g [95] (Figure 8). This can be ascribed to the great increase in quaternary ammonium salt functional groups, which can provide more adsorption sites.

### 4.3. Adsorption of Dyes

Dyes have caused numerous human health risks and environmental problems. ASFM fabricated with EBR technology has also been widely used for the adsorption of dyes. According to the difference in the charge, the progress of the corresponding ASFM is summarized in Table 4.

Acrylic acid (AAc) irradiation grafted xanthan gum hydrogel was fabricated for the adsorption of Rhodamine B (RhB) [96]; as the formed hydrogel has a porous structure with a huge surface area, thus ultrahigh adsorption capacity (2777.77 mg/g) of RhB was obtained with the hydrogel. PolyAAc grafted Artemisia sphaerocephala krasch gum (ASKG) was fabricated for the removal of methylene blue (MB) [97]; compared to the adsorption capacity (571.43 mg/g) of pure ASKG, the adsorption capacity reached 1654.9 mg/g for the polyAAc grafted one. EBR cross-linked chitosan-based double network hydrogel has as well been developed for the adsorption of MB [98], and the synthesized hydrogel had a large surface area of 96 m^2^/g. The maximum adsorption capacity of 246.12 mg/g was obtained with 60 kGy, and the hydrogel exhibited good regeneration performance. In our previous work, sodium 4-vinylbenzene sulfonate grafted bio-based loofah sponge (SSS-g-LFs) was fabricated for the removal of MB [99], the grafting rate was 40.45% with an SSS concentration of 20 wt%. Due to the formation of π-π stacking, electrostatic attraction, and hydrogen bond between the adsorbent and MB, a relatively high adsorption capacity was obtained. In our other work, SSS grafted magnetic chitosan microspheres were developed for the adsorption of MB [100], and an adsorption capacity of 191.60 mg/g was obtained for the CS-g-SPSS adsorbent at 140 kGy.

Zeolitic imidazolate framework-67 (ZIF-67) functionalized loofah was prepared through in situ growth with acrylic acid grafted loofah [101]. The synthesized adsorbent exhibited high adsorption capacity (433.90 mg/g) toward Congo red, which can be ascribed to the formation of pore adsorption, π-π stacking, hydrogen bond, and electrostatic attraction. Quaternary ammonium salts hydrogel (PQH) of polyquaternium-10 and polyethyleneglycol dimethacrylate was fabricated with radiated cross-linking [102], and the swelling ratio of the obtained hydrogel was 704%. The maximum adsorption capacity of PQH toward Coomassie blue was 617.28 mg/g, and the synthesized hydrogel exhibited a good regeneration performance. Methacryloxyethyl trimethyl ammonium chloride grafted kapok fiber (DKF) was developed for the selective adsorption of Indigo carmine (IC) [103]; the maximum capacity was 1011.30 mg/g, and the separation factor of IC/methyl orange reached 129.53. Quaternized sisal fiber (SF-DMC) was also fabricated for the removal of IC [104]; the isotherms adsorption fitted well with the Langmuir model, and the theoretical maximum capacity at different temperatures was in the range of 709.22~892.86 mg/g. 3-Diethylaminopropylamine functionalized bamboo fiber was prepared through radiation grafting [105] and used for the recycle adsorption of methyl orange (MO). The quaternized bamboo fiber (QBF) can efficiently remove the MO with a high adsorption capacity of 555.56 mg/g, and the capacity was unchanged after five adsorption-desorption cycles. Quaternized cotton linter (DMCCL) [106] and aminated cellulose microspheres (GFC) [107] were fabricated for the removal of MO, due to the quaternary ammonium anion that can form a strong electrostatic attraction with cationic MO, thus the adsorption capacity of DMCCL was much higher than GFC.

### 4.4. Adsorption of Drugs and Chemical Raw Materials

Drugs and chemical raw materials are other important pollutants in wastewater, which as well need to be removed. The ASFM prepared with EBR technology for their removal is summarized in Table 5.

**Figure 9:**
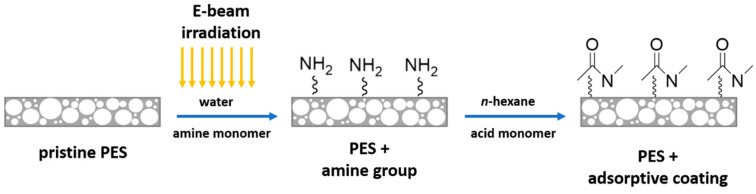
PES-based estradiol absorbents fabricated through co-irradiation grafting [109].

Ionic liquids modified tragacanth gum (TG-C_n_Br) hydrogel was fabricated via EBR cross-linking for the removal of diclofenac sodium [108]; due to the formation of π-π stacking, hydrogen bond, hydrophobicity interaction, and electrostatic attraction, the maximum adsorption capacity of 327.87 mg/g was obtained at 30 °C. In addition, the removal rate remained 94.55% after five recycles. Amidation-functionalized polyethersulfone (PES) membranes were developed for the adsorption of estradiol [109] (Figure 9), and compared to the PES membrane, the adsorption efficiency of the functionalized one increased from 30% to 60%. In addition, high water permeability was synthesized for the modified PES membrane, and no hole blockage was observed. Molecularly imprinted polymers (MIPs) were prepared through EBR cross-linking [110] and used for the recognition of sulfamethazine. The highest adsorption capacity of 31.00 mg/g was found at an irradiation dosage of 150 kGy, and the optimal imprinting factor was as high as 12.91.

**Figure 10:**
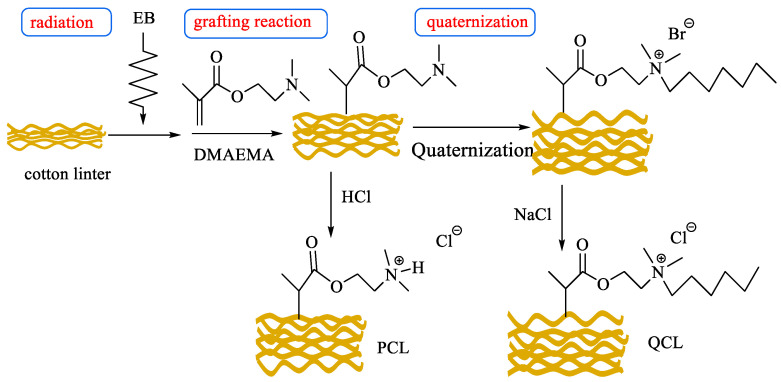
Cotton-based HA absorbents fabricated through pre-irradiation grafting [114].

Imidazolium ionic liquids ([C_8_VIm]Br) modified cellulose microsphere was fabricated for the efficient adsorption of *p*-Nitrophenol [111], the adsorption capacity increased to 327.87 mg/g due to the formation of a hydrogen bond, π-π stacking, and ion exchange. In addition, the fabricated adsorbent can selectively remove *p*-Nitrophenol from various real water. Nitrogen-doped active carbon fibers (ACFs) were fabricated via EBR and used for the removal of dimethyl methylyphosphonate (DMMP) [112]; compared to the raw active carbon fiber, the adsorption capacity of the ACFs increased by 14 times. EBR-aged microplastics have been developed for the removal of dibutyl phthalate [113]; due to the introduction of oxygen-containing functional groups after EBR, the adsorption capacity increases from 76.8 mg/g to 167.0 mg/g. This provides a new application of recycled plastics. Functionalized cotton linter was synthesized with EBR grafting of dimethylaminoethyl methacrylate; the quaternized adsorbent was used for the removal of humic acid (HA) [114] (Figure 10). Ascribed to the formation of strong electrostatic interaction, a high adsorption capacity of 333.00 mg/g was obtained.

### 4.5. Adsorption of Others

Besides the adsorption of water pollutants, the ASFM prepared with EBR technology has been used for the adsorption of biological substances and gases, and the most relevant examples are summarized in Table 6.

**Figure 11:**
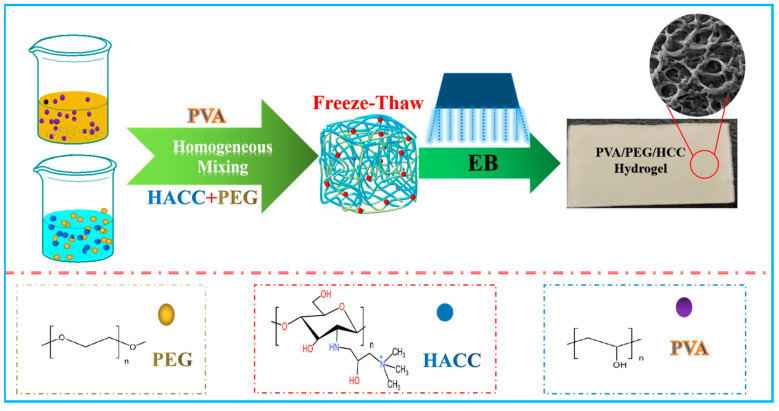
ctDNA absorbents fabricated through EBR cross-linking [115].

EBR cross-linked ternary composite hydrogel (PVA/PEG/HACC) was fabricated for the adsorption of ctNDA [115] (Figure 11), the maximum adsorption efficiency reached 89.00%. In addition, the adsorption efficiency remained at 71.39% after five recycles. Functionalized microsphere was synthesized through the coupling of grafted carboxymethyl chitin and dopamine, and used for the adsorption of blood cells [116]. With the help of this microsphere, the blood loss is reduced by 90%.

The triethylamine functionalized PE-PP fabric was prepared for the adsorption of CO_2_ [88] in a fixed bed column with a 10% CO_2_/N_2_ mixture, and an adsorption capacity of 45 mg/g was synthesized for the modified fabric. In addition, the saturated adsorbent can be desorpted at 80 °C. GMA grafted polyolefin membrane was fabricated for the selective permeability of CO_2_ [117], and the obtained PIM-1/gGMA membrane exhibited a separation factor of 48.3 for CO_2_/N_2_, with permeability CO_2_ of 1976 Barrer. Chitosan/lithium sulfonate double network hydrogel was synthesized for the capture of CO_2_ [118], due to the dipole–quadrup, Lewis base–acid, and coordination interaction between the hydrogel and CO_2_; a relatively high adsorption capacity of 67.90 mg/g was obtained. Ethanolamine-functionalized polyolefin nanofibers were fabricated with PP-g-GMA, and due to the high grafting yield of GMA (220%), the maximum adsorption capacity of the nanofibers reached 126.28 mg/g [119].

## 5. Mechanism of ASFM Toward Different Pollutants

The adsorption mechanism refers to various interactions between pollutants and adsorbents, which is the basis for the molecular design of ASFM [120,121,122]. Besides the porous structure of the matrix, a high-performance ASFM also requires a large number of functional groups that can form strong interactions with pollutant [123,124,125]. The characteristics of different pollutants and the interactions and structure of corresponding adsorbents are summarized in Table 7.

The metal elements always present as cations or anions in the water, which can be adsorbed through electrostatic interaction and chelating, thus an ideal adsorbent for metal ions should contain charged -COO^−^ or -NH_4_^+^, as well as lone pair electrons contained functional groups [126,127,128]. For the cationic metal species, the positive charge can easily form electrostatic interaction with a negative charged group like -COO^−^, and thus iminodiaetic acid functionalized ramie was designed for the removal of Pb^2+^ [68]. The anionic metal species such as Cr_2_O_7_^2−^ is favorable to form electrostatic interaction with positive charged groups like -NH_4_^+^; therefore, protonated amino-bamboo char was fabricated for the efficient adsorption of Cr(VI) [62]. The empty *d*-orbitals of transition metals and rare earth metals can form chelating interactions with heteroatoms, which contain lone pair electrons. Therefore, amino-phosphonic acid functionalized polystyrene microspheres were developed for the efficient removal of La(III), due to the strong coordination between the atoms of nitrogen, oxygen, and La(III) [74].

As the inorganic anions mainly form electrostatic attraction in the adsorption process, and the most common positive charged group is -NH_4_^+^, thus -NH_4_^+^ functionalized materials were designed for the removal of inorganic anions [129,130,131]. Based on this strategy, quaternized cotton linter fiber and quaternized bamboo sawdust were designed for the adsorption of PO_4_^3−^ [93,95].

The dyes are always aromatic compounds, which contain heteroatom and charged functional groups. Thus, the dyes not only can form π-π stacking, but also can easily form electrostatic attraction and hydrogen bonds [132,133,134]. Based on the electrostatic attraction, π-π stacking, and hydrogen bond between cationic dye and adsorbents, sodium 4-vinylbenzene sulfonate grafted loofah sponge was fabricated for the efficient removal of cationic methylene blue [99]. Based on the same strategy, zeolitic imidazolate framework-67 functionalized loofah was developed for the adsorption of anionic Congo red [101].

The drugs are similar to dyes, which can easily form π-π stacking and hydrogen bonds with adsorbents. In addition, electrostatic attraction can be formed between the ionic drugs and adsorbents [135,136,137]. For the efficient removal of diclofenac sodium, ionic liquids functionalized tragacanth gum hydrogel was fabricated. Due to the strong interaction between quaternized aromatic ionic liquids and dichofenac sodium, the high removal efficiency was achieved for the fabricated adsorbent [108].

The CO_2_ is a Lewis acid, which can react with alkaline compounds, thus the adsorbents of CO_2_ always contain -NH_2_ and -NHR [138,139,140]. Radiation-initiated chitosan composite hydrogel was fabricated for the capture of CO_2_, and due to the reversible reaction between CO_2_ and nitrogen-containing groups, high adsorption capacity was achieved for the fabricated composite hydrogel [118].

## 6. Conclusions and Future Prospects

To meet the requirements of high-quality and sustainable development, clean and efficient EBR technology has been successfully used in the preparation of ASFM for pollutant removal. There are three methods of EBR technology for the preparation of ASFM, namely pre/co-radiation grafting, irradiation cross-linking, and irradiation-induced structure transformation. The ASFMs fabricated by EBR technology are diverse, including functional groups-grafted fibers, fabrics, membranes, particles, and nanomaterials. In addition, cross-linked hydrogels, MIP, COF, and structure-transformed microplastics, MOF, and clay were developed for the removal of pollutants. The ASFM prepared with EBR technology has been extensive applied for the removal of metal ions, inorganic anions, dyes, drugs, chemical raw materials, biological substances, and gases, many adsorbents with excellent adsorption performance and good regeneration behavior were obtained. The removal of pollutants depends on the interaction between the pollutants and the adsorbents; thus, the porous structure and functional groups are the most important factors for a high-performance adsorbent.

Though great progress has been made for the ASFM fabricated with EBR technology and excellent adsorption performance was achieved for the prepared adsorbents, there are still some issues that need to be addressed.

(1) The variety of ASFM fabricated with EBR technology should be further expanded. With the development of the industry, many new pollutants such as flame retardants, surfactants, nitrogen oxides, and sulfur oxides were emitted into the environment, but no corresponding adsorbents were prepared with EBR technology at this stage.
(2) The selectivity of the synthesized ASFM should be improved. It is necessary to selectively remove pollutants from products in many cases, where adsorbents with selectivity are needed. Thus, MIPs fabricated with EBR technology should developed.
(3) Though the static adsorption performance of the obtained adsorbents is excellent, there are few reports about the dynamic adsorption performance, which is far behind the industrial application.
(4) There are few investigations about the quantitative investigation of the interaction between pollutants and adsorbents, which is not beneficial for the precise design and synthesis of adsorbents.
(5) Most of the ASFM prepared with EBR technology stays in the laboratory stage, and there is almost no industrial production and application. Thus, the process of industrialization needs to be accelerated.

## Figures and Tables

**Table 1:** ASFM prepared with EBR technology for metal cations adsorption.

Metal Type	Cations	ASFM	Adsorption Performance	EBR Technology	Ref.
Heavy metal	Co^2+^	Sheep wool	29.96 mg/g, 153 kGy	structure transformation	[66]
	Cu^2+^	TC4A/PAA/diatomite	123.30 mg/g, 90 kGy, 30 kGy/pass	pre-irradiation	[67]
	Pb^2+^	Ramie-GMA-IDA	225.71 mg/g, 200 kGy, 20 kGy/pass, pH = 5.0	co-irradiation	[68]
	Cd^2+^	LFs-PGMA-IDAA	169.81 mg/g, 240 kGy, 5 kGy/pass, pH = 5.0	co-irradiation	[69]
	Hg^2+^	CysMC and PMC	264.55 and 169.49 mg/g, 30 kGy, 10 kGy/pass	pre-irradiation	[70]
	Ga^3+^	MCC-g-GMA-TA	26.55 mg/g, 30 kGy, 10 kGy/pass, pH = 3.0	pre-irradiation	[71]
	In^3+^	MCC-g-GMA-TA	35.63 mg/g, 30 kGy, 10 kGy/pass, pH = 3.0	pre-irradiation	[71]
Precious metal	Ag^+^	CysMC	66.67 mg/g, 30 kGy, 5 kGy/pass	pre-irradiation	[72]
	Ag^+^	2-AMP	110.5 mg/g, 30 kGy, pH = 4.9	pre-irradiation	[73]
Rare earth	La^3+^	PS-PGMA-DETA	288.69 mg/g, 240 kGy, 10 kGy/pass, pH = 6.0	co-irradiation	[74]
	Ce^3+^	PMMA-PGMA-PEI	189.81 mg/g, 80 kGy, 20 kGy/pass, pH = 6.0	co-irradiation	[75]

**Table 2:** ASFM prepared with EBR technology for metal-containing anions adsorption.

Metal Type	Metal Anions	ASFM	Adsorption Performance	EBR Technology	Ref.
Cr	Cr_2_O_7_^2−^	Microplastic PP	9.3 mg/g, 500 kGy	structure transformation	[76]
	Cr_2_O_7_^2−^	PE/PP fiber	38.43 mg/g, 100 kGy	pre-irradiation	[77]
	Cr_2_O_7_^2−^	TiO_2_-gGMA/NMG	81.8%, 60 kGy, 30 kGy/pass, pH = 3.0	co-irradiation	[78]
	Cr(SO_4_)_2_^−^	Sheep wool	30 mg/g, 24 kGy	structure transformation	[79]
Au	AuCl_4_^−^	MCC-g-GMA-Cys	714.28 mg/g, 10 kGy, 5 kGy/pass	pre-irradiation	[80]
	AuCl_4_^−^	n-AMPRs	537.53 mmol/g, 10 kGy, 10 kGy/pass	pre-irradiation	[81]
	AuCl_4_^−^	MCC-g-GMA-Amino	769.23 mg/g, 10 kGy, 5 kGy/pass	pre-irradiation	[82]
Radioactive metal	ReO_4_^−^	MCC-g-Nucleobases	23.43~194.0 mg/g	pre-irradiation	[83]
	ReO_4_^−^	AGfC	264.55 mg/g, 30 kGy, 30 kGy/pass	pre-irradiation	[84]
	ReO_4_^−^	MCC-g-Ionic Liquids	230.41 mg/g, 160 kGy, 20 kGy/pass	co-irradiation	[85]
	ReO_4_^−^/Mo_7_O2_4_^6−^	MCGA	134.05/27.96 mg/g, 50 kGy, 5 kGy/pass	pre-irradiation	[86]

**Table 3:** ASFM prepared with EBR technology for inorganic anions adsorption.

Anions	ASFM	Adsorption Performance	EBR Technology	Ref.
F^−^	CMC + MWCCNT-ZrOCl_2_	36.66 mmol/g, 10 kGy, 5 kGy/pass	cross-linking	[87]
BO_3_^3−^	PE-PP-g-MG	23.78 mg/g, 50 kGy, 10 kGy/pass	pre-irradiation	[88]
Cl_3_CCOO^−^	Nano-oxide@MCC	83.27%, 60 kGy, 10 kGy/pass	pre-irradiation	[89]
ClO_2_^−^	NWF-g-DMC@Al_2_O_3_	89.00%, 100 kGy, 20 kGy/pass	pre-irradiation	[90]
PO_4_^3−^	MCC-g-AM/EDA/PA-Fe(II)	89.70%, 30 kGy, 10 kGy/pass	pre-irradiation	[91]
PO_4_^3−^	QCL	36.13 mg/g, 60 kGy, 10 kGy/pass	pre-irradiation	[92]
PO_4_^3−^	CL-g-GMA-Q	152.44 mg/g, 50 kGy, 10 kGy/pass	pre-irradiation	[93]
PO_4_^3−^	DMCBF	67.48 mg/g, 50 kGy, 10 kGy/pass	pre-irradiation	[94]
PO_4_^3−^	BS-GMA-PEHA-Q	152.21 mg/g, 70 kGy, 10 kGy/pass	co-irradiation	[95]

**Table 4:** ASFM fabricated with EBR technology for dye adsorption.

Dye Type	Dyes	ASFM	Adsorption Performance	EBR Technology	Ref.
Cationic	Rhodamine B	AA-g-XGH	2777.77 mg/g, 70 kGy, 10 kGy/pass	cross-linking	[96]
	Methylene blue	ASKG hydrogel	1654.9 mg/g, 70 kGy, 10 kGy/pass	cross-linking	[97]
	Methylene blue	CTS hydrogel	246.12 mg/g, 60 kGy, 10 kGy/pass	cross-linking	[98]
	Methylene blue	SSS-g-LFs	275.60 mg/g, 200 kGy, 20 kGy/pass	co-irradiation	[99]
	Methylene blue	CS-g-SPSS	191.60 mg/g, 140 kGy, 20 kGy/pass	co-irradiation	[100]
Anionic	Congo red	MOF-LFs	433.90 mg/g, 60 kGy, 10 kGy/pass	co-irradiation	[101]
	Coomassie blue	PQH hydrogels	617.28 mg/g, 80 kGy, 10 kGy/pass	cross-linking	[102]
	Indigo carmine	DKF	1011.30 mg/g, 60 kGy, 10 kGy/pass	pre-irradiation	[103]
	Indigo carmine	SF-DMC	892.86 mg/g, 60 kGy, 10 kGy/pass	co-irradiation	[104]
	Methyl orange	QBF	555.56 mg/g, 60 kGy, 10 kGy/pass	co-irradiation	[105]
	Methyl orange	DMCCL	645.16 mg/g, 70 kGy, 10 kGy/pass	co-irradiation	[106]
	Methyl orange	GFC	63.25 mg/g, 20 kGy, 5 kGy/pass	pre-irradiation	[107]

**Table 5:** ASFM prepared with EBR technology for drugs and chemical raw materials adsorption.

Type	Compounds	ASFM	Adsorption Performance	EBR Technology	Ref.
Drugs	Diclofenac sodium	TG-C_n_Br hydrogel	327.87 mg/g, 80 kGy, 10 kGy/pass	Cross-linking	[108]
	Estradiol	Amidated-PES	>60%, 200 kGy	Co-irradiation	[109]
	Sulfamethazine	MIPs	31.00 mg/g, 150 kGy	Cross-linking	[110]
Chemical raw materials	*p*-Nitrophenol	MCC-[C_8_VIm]Br	327.87 mg/g, 80 kGy	Co-irradiation	[111]
	DMMP	ACF	36.6 mg/g, 200 kGy, 10 kGy/pass	Structure transformation	[112]
	Dibutyl phthalate	Microplastics PE	167.0 mg/g, 350 kGy	Structure transformation	[113]
	Humic acid	QCL	333.00 mg/g, 60 kGy, 10 kGy/pass	Pre-irradiation	[114]

**Table 6:** ASFM prepared with EBR technology for other substance adsorption.

Type	Compounds	ASFM	Adsorption Performance	EBR Technology	Ref.
Biological substance	ctDNA	PVA/PEG/HACC	89.00%, 25 kGy, 5 kGy/pass	cross-linking	[115]
	Blood cell	CCM-g-AA@DA	90%, 25 kGy, 5 kGy/pass	co-irradiation	[116]
Gases	CO_2_	PE-PP-g-MG	45 mg/g, 50 kGy, 10 kGy/pass	pre-irradiation	[88]
	CO_2_	PIM-1/gGMA	1976 Barrer, 80 kGy	co-irradiation	[117]
	CO_2_	CS/LS hydrogel	67.90 mg/g, 40 kGy, 10 kGy/pass	cross-linking	[118]
	CO_2_	PP nanofibers	126.28 mg/g, 180 kGy	pre-irradiation	[119]

**Table 7:** Molecular design of ASFM.

Pollutants	Characteristics	Interactions	Functional Groups	Refs.
Metal ions	Positive/negative, d-orbital	Electrostatic, chelating	-COO^−^/-NH_4_^+^, -OH, -NH_2_	[126,127,128]
Inorganic anions	Negative	Electrostatic attraction	-NH_4_^+^	[129,130,131]
Dyes	Positive/negative, aromatic, heteroatom	Electrostatic, π-π stacking, hydrogen bond	-COO^−^/-NH_4_^+^, -Ar, -OH, -NH_2_	[132,133,134]
Drugs	Aromatic, heteroatom	π-π Stacking, hydrogen bond	-Ar, -OH, -NH_2_	[135,136,137]
CO_2_	Lewis acid	Base–acid pair	-NH_2_, -NHR	[138,139,140]

## Data Availability

No new data were created or analyzed in this study. Data sharing is not applicable to this article.

## References

[1] Sullivan S.M.P., Rains M.C., Rodemald A.D., Buzbee W.W., Rosemond A.D. (2020). Distorting science, putting water at risk. Science.

[2] Sala E., Mayorga J., Bradley D., Cabral R.B., Atwood T.B., Auber A., Cheung W., Costello C., Ferretti F., Friedlander A.M. (2021). Protecting the global ocean for biodiversity, food and climate. Nature.

[3] Dehkordi M.M., Nodeh Z.P., Dehkordi K.S., Salmanvandi H., Khorjestan R.R., Ghaffarzadeh M. (2024). Soil, air, and water pollution from mining and industrial activities: Sources of pollution, environmental impacts, and prevention and control methods. Results Eng..

[4] Patel M., Kumar R., Kishor K., Mlsna T., Pittman C.U., Mohan D. (2019). Pharmaceuticals of emerging concern in aquatic systems: Chemistry, occurrence, effects, and removal methods. Chem. Rev..

[5] Lu Y.Y., Ma T.T., Lan Q.W., Liu B.Y., Liang X.Q. (2024). Single entity collision for inorganic water pollutants measurements: Insights and prospects. Water Res..

[6] Sewify I.M., Ma S.Q. (2024). Recent development of metal-organic frameworks for water purification. Langmuir.

[7] Adesibikan A.A., Emmanuel S.S., Olawoyin C.O., Ndungu P. (2024). Cellulosic metallic nanocomposites for photocatalytic degradation of persistent dye pollutants in aquatic bodies: A pragmatic review. J. Organmet. Chem..

[8] Chen Z.Y., Petetin H., Turrubiates R.F.M., Achebak H., Pando C.P.G., Ballester J. (2024). Population exposure to multiple air pollutants and its compound episodes in Europe. Nat. Commun..

[9] Yan X.J., Ying Y.F., Li K.K., Zhang Q., Wang K.Y. (2024). A review of mitigation technologies and management strategies for greenhouse gas and air pollutant emissions in livestock production. J. Environ. Manag..

[10] Nandi B.P., Jain G.S.A., Tayal D.K. (2024). Evolution of neural network to deep learning in prediction of air, water pollution and its Indian context. Int. J. Environ. Sci. Tech..

[11] Jiang S., Li E., Wei Y.M., Yan X.X., He R.F., Banny E.T., Xin Z. (2024). Measurement and influencing factors of carbon emission effciency based on the dual perspectives of water pollution and carbon neutrality. Sci. Total Environ..

[12] Naseem K., Aziz A., Tahir M.H., Ameen A., Ahmad A., Ahmad K., Arif M., Hassan W., Najeeb J., Rao E. (2024). Biogenic synthesized nanocatalysts and their potential for the treatment of toxic pollutants: Environmental remediation, a review. Int. J. Environ. Sci. Tech..

[13] Yuan L., Qi Y.Z., He W.J., Wu X., Kong Y., Ramsey T.S., Degefu D.M. (2024). A differential game of water pollution management in the trans-jurisdictional river basin. J. Clean. Prod..

[14] Chabalala M.B., Mothudi B.M., Ntsendwana B. (2024). MOF based nanocomposites for photocatalytic degradation of pollutants in water: A critical review. J. Photoch. Photobio. A.

[15] Liu H.L., Tang S.F., Wang Z.B., Zhang Q.R., Yuan D.L. (2024). Organic cocatalysts improved Fenton and Fenton-like processes for water pollution control: A review. Chemosphere.

[16] Zhou Q.L., Lei P.L., Cheng S.Y., Wang H., Dong W., Pan X.H. (2024). Recent progress in magnetic polydopamine composites for pollutant removal in wastewater treatment. Int. J. Biol. Macromol..

[17] Xie S.J., Yan J., Alhassan S.I., Huang L., Sio W.H., Zeng Z., Zhang H.G. (2024). Application of metal nitrides in catalysis and adsorption of pollutants in water. J. Environ. Chem. Eng..

[18] Uddin M.K. (2017). A review on the adsorption of heavy metals by clay minerals, with special focus on the past decade. Chem. Eng. J..

[19] Shen J., Kumar A., Wahiduzzaman M., Barpaga D., Maurin G., Motkuri R.K. (2024). Engineered nanoporous frameworks for adsorption cooling applications. Chem. Rev..

[20] Du J.F., Dong Z., Xie C., Yang X., Yang L., Zhao L., Zhai M.L. (2016). Application of radiation grafting technique in preparation of adsorption materials. Acta Polym. Sin..

[21] Botella E.P., Valencia S., Rey F. (2022). Zeolites in adsorption processes: State of the art and future prospects. Chem. Rev..

[22] Biswas G., Kumari M., Ahhikari K., Dutta S. (2017). A critical review on occurrence of fluoride and its removal through adsorption with an emphasis on natural minerals. Curr. Pollut. Rep..

[23] Tamjidi S., Ameri A. (2020). A review of the application of sea material shells as low cost and effective bio-adsorbent for removal of heavy metals from wastewater. Environ. Sci. Pollut. Res..

[24] Miao P.P., Gao J., Han X.B., Zhao Y., Chen T. (2024). Adsorption of levofloxacin onto graphene oxide/chitosan composite aerogel microspheres. Gels.

[25] Mohammadabadi S.I., Javanbakht V. (2020). Lignin extraction from barley straw using ultrasound-assisted treatment method for a lignin-based biocomposite preparation with remarkable adsorption capacity for heavy metal. Int. J. Biol. Macromol..

[26] Bekchanov D., Mukhamediev M., Yarmanov S., Lieberzeit P., Mujahid A. (2024). Functionalizing natural polymers to develop green adsorbents for wastewater treatment applications. Carbohyd. Polym..

[27] Zhu X.Y., Hou X.L., Ma B.M., Xu H.L., Yang Y.Q. (2019). Chitosan/gallnut tannins composite fiber with improved tensile, antibacterial and fluorescence properties. Carbohyd. Polym..

[28] Duan Y.Q., Freyburger A., Kunz W., Zollfrank C. (2018). Lignin/chitin films and their adsorption characteristics for heavy metal ions. ACS Sustain. Chem. Eng..

[29] Yang K., Xing B.S. (2010). Adsorption of organic compounds by carbon nanomaterials in aqueous phase: Polanyi theory and its application. Chem. Rev..

[30] Li J., Wang X.X., Zhao G.X., Chen C.L., Chai Z.F., Alsaedi A., Hayat T., Wang X.K. (2018). Metal-organic framework-based materials: Superior adsorbents for the capture of toxic and radioactive metal ions. Chem. Soc. Rev..

[31] Zhang P., He M.M., Teng W., Li F.K., Qiu X.Y., Li K.X., Wang H. (2024). Ordered mesoporous materials for water pollution treatment: Adsorption and catalysis. Green Energy Environ..

[32] Singh B.P., Tyagi L., Vikal S., Tyagi S., Tyagi D., Rani M., Sharma K., Shukla G., Shanker U., Gautam Y.K. (2024). Spotlighting graphene-based nanomaterials for the mitigation of hazardous water pollutants: A review. Inorg. Chem. Commun..

[33] Wang Q., Tang S.F., Zhang Y.Q., Lai C.J.S. (2024). Recent development of natural polysaccharide-modified biochar on adsorption of pollutants from wastewater: Preparation, characterization, mechanisms and applications. Sep. Purif. Technol..

[34] Shaghaleh H., Hamoud Y.A., Sun Q. (2024). Functionalized nanocellulose nanocomposite hydrogels for soil and water pollution prevention, remediation, and monitoring: A critical review on fabrication, application properties, and potential mechanisms. J. Environ. Chem. Eng..

[35] Teng H.J., Xia T., Li C., Guo J.Z., Chen L., Wu C.Z., Li B. (2023). Facile solvent-free radical polymerization to prepare itaconate-functionalized hydrochar for effcient sorption of methylene blue and Pb(II). Bioresource Technol..

[36] Zhang Y.N., Guo J.Z., Wu C.Z., Huan W.W., Chen L., Li B. (2022). Enhanced removal of Cr(VI) by cation functionalized bamboo hydrochar. Bioresource Technol..

[37] Grasselli M., Smolko E.E. (2022). Designing protein adsorptive materials by simultaneous radiation-induced grafting polymerization: A review. Radiat. Phys. Chem..

[38] Barsbay M., Guven O. (2019). Surface modification of cellulose via conventional and controlled radiation-induced grafting. Radiat. Phys. Chem..

[39] Wojnarovits L., Foldvary C.M., Takacs E. (2010). Radiation-induced grafting of cellulose for adsorption of hazardous water pollutants: A review. Radiat. Phys. Chem..

[40] Dong Z., Wang Y., Wen D., Peng J., Zhao L., Zhai M.L. (2022). Recent progress in environmental applications of functional adsorbent prepared by radiation techniques: A review. J. Hazard. Mater..

[41] Torkaman R., Maleki F., Gholami M., Mostaedi M.T., Asadollahzadeh M. (2021). Assessing the radiation-induced graft polymeric adsorbents with emphasis on heavy metals removing: A systematic literature review. J. Water Process Eng..

[42] Nasef M.M., Guven O. (2012). Radiation-grafted copolymers for separation and purification purposes: Status, challenges and future directions. Prog. Polym. Sci..

[43] Pavlov Y.S., Petrenko V.V., Alekseev P.A., Bystrov P.A., Souvorova O.V. (2022). Trends and opportunities for the development of electron-beam energy-intensive technologies. Radiat. Phys. Chem..

[44] Wali A.S., Kumar S., Khan D. (2023). A review on recent development and application of radiation curing. Mater. Today Proceed..

[45] Ahmed M.S., Islam M., Hasan M.K., Nam K.W. (2024). A Comprehensive review of radiation-induced hydrogels: Synthesis, properties, and multidimensional applications. Gels.

[46] Elmaaty T.A., Okubayashi S., Elsisi H., Abouelenin S. (2022). Electron beam irradiation treatment of textiles materials: A review. J. Polym. Res..

[47] Zhang L.C., Liu Y.J., Li S.J., Hao Y.L. (2018). Additive manufacturing of titanium alloys by electron beam melting: A review. Adv. Eng. Mater..

[48] Yoo S.H., Park S., Park Y., Lee D., Joh H.I., Shi I., Lee S. (2017). Facile method to fabricate carbon fibers from textile-grade polyacrylonitrile fibers based on electron-beam irradiation and its effect on the subsequent thermal stabilization process. Carbon.

[49] Xiong G.W., Jia J., Zhao L.L., Liu X.Y., Zhang X.L., Liu H., Zhou W.J. (2021). Non-thermal radiation heating synthesis of nanomaterials. Sci. Bull..

[50] Conard S., Kumar P., Xue F., Ren L.M., Henning S., Xiao C.H., Mkhoyan K.A., Tsapstis M. (2018). Controlling dissolution and transformation of zeolitic imidazolate frameworks by using electron-beam-induced amorphization. Angew. Chem..

[51] Zhang M.X., Chen J.C., Zhang S.T., Zhou X.Q., He L.W., Sheridan M.V., Yuan M.J., Zhang M.J., Chen L., Dai X. (2020). Electron beam irradiation as a general approach for the rapid synthesis of covalent organic frameworks under ambient conditions. J. Am. Chem. Soc..

[52] Arunachalam P., Lalitha R., Vanniarajan C., Souframanien J. (2022). Comparative analysis of gamma rays and electron beam in altering rice (*Oryza sativa* L.) grain size. Indian J. Genet..

[53] Gotzmann G., Portillo J., Wronski S., Kohl Y., Gorjup E., Schuck H., Rogner F.H., Muller M., Chaberny I.F., Schonfelder J. (2018). Low-energy electron-beam treatment as alternative for on-site sterilization of highly functionalized medical products-A feasibility study. Radiat. Phys. Chem..

[54] Lu J., Sui X.M., Novoselov K.S., Huang P.R., Xu F., Sun L.X. (2024). Electron beam-assisted synthesis and modifcation of electrode/separator materials for lithium-ion batteries: Progress and prospects. Coordin. Chem. Rev..

[55] Lim K.L., Wong C.Y., Wong W.Y., Loh K.S., Selambakkannu S.S., Othman N.A.F., Yang H. (2021). Radiation-grafted anion-exchange membrane for fuel cell and electrolyzer applications: A mini review. Membranes.

[56] Meibodi M.E., Parsaeian M.R., Amraei R., Banaei M., Anvari F., Tahami S.M.R., Vakhshoor B., Mehdizadeh A., Nejad N.F., Shirmardi S.P. (2016). An experimental investigation of wastewater treatment using electron beam irradiation. Radiat. Phys. Chem..

[57] Liu X.Y., Wang J.L. (2024). Decolorization and degradation of various dyes and dye-containing wastewater treatment by electron beam radiation technology: An overview. Chemosphere.

[58] Haque S.N., Bhuyan M.M., Heong J.H. (2024). Radiation-induced hydrogel for water treatment. Gels.

[59] Shen Y.S., Miao P.P., Liu S.C., Gao J., Han X.B., Zhao Y., Chen T. (2023). Preparation and application progress of imprinted polymers. Polymers.

[60] Zhang J., Wang J.H., Zhu F.K., Mao P., Wu Z.Y., Hong K. (2022). Dispersing bentonite by electron beam irradiation and its adsorption performance of Cr(VI) in the aqueous solution. Water Air Soil Pollut..

[61] Chen L., Shao H.Y., Ren Y.F., Mao C.K., Chen K., Wang H.Y., Jing S.T., Xu C.W., Xu G. (2024). Investigation of the adsorption behavior and adsorption mechanism of pollutants onto electron beam-aged microplastics. Sci. Total Environ..

[62] Zhao Y., Gao J., Liang T., Chen T., Han X.B., Hu G.W., Li B. (2023). Efficient removal of Cr(VI) by protonated amino-bamboo char prepared via radiation grafting: Behavior and mechanism. Sustainability.

[63] Cui Y., Chen X.B., Wang Y.C., Peng J., Zhao L., Du J.F., Zhai M.L. (2019). Amphoteric ion exchange membranes prepared by preirradiation-induced emulsion graft copolymerization for vanadium redox flow battery. Polymers.

[64] Manaila E., Cracium G., Ighigeanu D., Cimpeanu C., Barna C., Fugaru V. (2017). Hydrogels synthesized by electron beam irradiation for heavy metal adsorption. Materials.

[65] Miao P.P., Sang Y.N., Gao J., Han X.B., Zhao Y., Chen T. (2023). Adsorption and recognition property of tyrosine molecularly imprinted polymer prepared via electron beam irradiation. Polymers.

[66] Branisa J., Jomova K., Lapcik L., Porubska M. (2021). Testing of electron beam irradiated sheep wool for adsorption of Cr(III) and Co(II) of higher concentrations. Polym. Test..

[67] Tang X.Q., Hu G.W., Chen Z.Y., Qian L.B., He C.Q., Chen T., Gao J., Zhao Y., Han X.B. (2024). High adsorption of Cu(II) in novel thiacalix[4]arene/acrylic polymer/diatomite fast fabricated by electron beam irradiation: Controllable microstructures, adsorption performance and mechanism. Chem. Eng. J..

[68] Zhao Y., Zheng Y., Chu C.K., Liang T., Tian Y.Y., Chen L.F., Li B., Gao J., Chen T. (2024). High adsorption capacity of Pb^2+^ by iminodiacetic acid functionalized ramie via radiation grafting. J. Saudi Chem. Soc..

[69] Zhao Y., Chen T., Liang T., Yang J.Y., Song X.F., Yang X.J., Li Y.S., Liu Y. (2023). Radiation synthesis of IDAA functionalized loofah sponge for enhanced removal of Pb(II) and Cd(II): Behavior and mechanism investigation. J. Mol. Liq..

[70] Du J.F., Ye Q., Dong Z., Yang X., Zhao L. (2021). Selective adsorption of mercury(II) ions from aqueous solution by two kinds of modifed cellulose microsphere. Desalin. Water Treat..

[71] Du J.F., Zhang M.M., Dong Z., Yang X., Zhao L. (2022). Facile fabrication of tannic acid functionalized microcrystalline cellulose for selective recovery of Ga(III) and In(III) from potential leaching solution. Sep. Purif. Technol..

[72] Dong Z., Yang X., Pan Q., Ao Y.Y., Du J.F., Zhai M.L., Zhao L. (2020). Performance and mechanism of selective adsorption of silver to L-cysteine functionalized cellulose microsphere. Cellulose.

[73] Yang X., Dong Z., Zhang M.M., Du J.F., Zhao L. (2020). Selective recovery of Ag(I) using a cellulose-based adsorbent in high saline solution. J. Chem. Eng. Data.

[74] Chen T., Sun N., Zhao Y., Gao J., Hu G.W., Han X.B., Tian Y.Y., Chen L.F., Huang G.B., Li B. (2022). Removal of La(III) by amino-phosphonic acid functionalized polystyrene microspheres prepared via electron beam irradiation. J. Saudi Chem. Soc..

[75] Zhao Y., Liang T., Miao P.P., Chen T., Han X.B., Hu G.W., Gao J. (2022). Green preparation of aminated magnetic PMMA microspheres via EB irradiation and its highly efficient pptake of Ce(III). Materials.

[76] Chen L., Xie N., Yuan S.N., Shao H.Y. (2024). Adsorption mechanism of hexavalent chromium on electron beam-irradiated aged microplastics: Novel aging processes and environmental factors. Chemosphere.

[77] Limsuwan Y., Hemvichian K., Hoshina H., Chen J.H., Shimoyama Y., Amada H., Seko N., Pongprayoon T. (2024). PE/PP fbrous adsorbents with bifunctional groups of tertiary amine and phosphate prepared by electron beam induced co-grafting for heavy metal adsorption of both cationic and anionic forms in acidic conditions. Polymer.

[78] Li Y.S., Li T.T., Song X.F., Yang J.Y., Liu G., Qin J.T., Dong Z.B., Chen H.G., Liu Y. (2020). Enhanced adsorption-photocatalytic reduction removal for Cr (VI) based on functionalized TiO_2_ with hydrophilic monomers by pre-radiation induced grafting-ring opening method. Appl. Surf. Sci..

[79] Porubska M., Koosova K., Branisa J. (2023). Competitive cation adsorption on electron-irradiated sheep wool changes the fitting of adsorption isotherms for single-component solutions. Processes.

[80] Yang X., Pan Q., Ao Y.Y., Du J.F., Dong Z., Zhai M.L., Zhao L. (2020). Facile preparation of L-cysteine–modified cellulose microspheres as a low-cost adsorbent for selective and efficient adsorption of Au(III) from the aqueous solution. Environ. Sci. Pollut. Res..

[81] Dong Z., Liu J.Z., Yuan W.J., Yi Y.P., Zhao L. (2016). Recovery of Au(III) by radiation synthesized aminomethyl pyridine functionalized adsorbents based on cellulose. Chem. Eng. J..

[82] Hao F.L., Du J.F., Peng L.F., Zhang M.M., Dong Z., Shen Y.B., Zhao L. (2023). Selective and effective gold recovery from printed circuit boards and gold slag using amino-acid-functionalized cellulose microspheres. Polymers.

[83] Li W.K., Zhang M.M., Peng L.F., Du J.F., Hua R., Zhao L. (2023). Selective recovery of Re(VII) by nucleobases functionalized cellulose microspheres from the simulated uranium ore leaching solution. Int. J. Biol. Macromol..

[84] Du J.F., Dong Z., Wen D., Yang X., Zhao M.L., Hua R., Zhao L. (2022). Selective recovery of rhenium from the simulating leaching solutions of uranium ore by amino guanidine functionalized microcrystalline cellulose microsphere. J. Mol. Liq..

[85] Peng L.F., Zhang M.M., Dong Z., Du J.F., Li W.K., Zhao L. (2024). Effcient and selective adsorption of TcO_4_^−^/ReO_4_^−^ by n-alkyl- imidazolium ionic liquids functionalized cellulose microspheres and their application in simulated Beishan groundwater. J. Mol. Liq..

[86] Zhang M.M., Du J.F., Dong Z., Qi W., Zhao L. (2022). Recovery and separation of Mo(VI) and Re(VII) from Mo-Re bearing solution by gallic acid-modifed cellulose microspheres. Sep. Purif. Technol..

[87] Zeng Z.K., Xu K., Zhang F.X., Yang X., Du J.F., Dong Z., Bao Q., Zhao L. (2024). Facile preparation of zirconium(IV) immobilized carboxymethyl cellulose/multiwalled carbon nanotubes gels by radiation technique and its selective fluoride removal. Int. J. Biol. Macromol..

[88] Nasef M.M., Ting T.M., Abbasi A., Moghaddam A.L., Alinezhad S.S., Hashim K. (2016). Radiation grafted adsorbents for newly emerging environmental applications. Radiat. Phys. Chem..

[89] Fu L.L., Wang Z.J., Liu K., Tang D.X., Yang J.Y., Chen H.Q., Li Y.S. (2024). Radiation preparation of nano-oxide@microcrystalline cellulose and its adsorption and removal of trichloroacetic acid. J. Radiat. Res. Radiat. Process.

[90] Rao L., Liu D.L., Fu L.L., Wang Z.J., Chen H.Q., Li Y.S. (2023). Preparation of NWF-g-DMC@Al_2_O_3_ adsorbent by preradiation grafting-embedding and its application for removal of chlorite. J. Radiat. Res. Radiat. Process.

[91] Zhao Y., Yang J.Y., Li T.T., Liu G., Wang Y.Y., Jiang Y., Wang Z.X., Zhang F.F., Li Y.S., Liu Y. (2021). Controlled preparation of a MCC-g-AM/EDA/PA loaded Fe(III) adsorbent by the pre-radiation grafting method and its application for the adsorption removal of phosphate. RSC Adv..

[92] Du J.F., Dong Z., Yang X., Zhao L. (2020). Radiation grafting of dimethylaminoethyl methacrylate on cotton linter and subsequent quaternization as new eco-friendly adsorbent for phosphate removal. Environ. Sci. Pollut. Res..

[93] Du J.F., Dong Z., Lin Z.Y., Yang X., Zhao L. (2019). Radiation synthesis of pentaethylene hexamine functionalized cotton linter for effective removal of phosphate: Batch and dynamic flow mode studies. Materials.

[94] Du J.F., Dong Z., Yang X., Zhao L. (2021). Fabrication of bamboo fiber-based adsorbent by radiation grafting and rapid adsorption of phosphate. J. Radiat. Res. Radiat. Process.

[95] Du J.F., Xiong H.H., Dong Z., Yang X., Zhao L., Yang J. (2021). Ethylenediamine and pentaethylene hexamine modified bamboo sawdust by radiation grafting and their adsorption behavior for phosphate. Appl. Sci..

[96] Du J.F., Yang X., Xiong H.H., Dong Z., Wang Z.W., Chen Z.Y., Zhao L. (2021). Ultrahigh adsorption capacity of acrylic acid-grafted xanthan gum hydrogels for Rhodamine B from aqueous solution. J. Chem. Eng. Data.

[97] Du J.F., Fan D.C., Yang X., Dong Z., Zhao L. (2023). Facile fabrication of Artemisia sphaerocephala krasch gum hydrogels by radiation induced cross-linking polymerization and enhanced ultrahigh adsorption for methylene blue. Int. J. Biol. Macromol..

[98] Du W.J., Fan J.X., Ma R., Yang G., Liu J.Q., Zhang S.F., Chen T. (2021). Radiation-initiated chitosan-based double network hydrogel: Synthesis, characterization, and adsorption of methylene blue. J. Appl. Polym. Sci..

[99] Zhao Y., Chen T., Song X.F., Yang J.Y., Wang Y.Y., Liu Y.S., Liu Y. (2022). Green synthesis of loofah-based biosorbent via radiation grafting for effective removal of methylene blue. Arab. J. Chem..

[100] Chen T., Zhao Y., Sang Y.N., Tang M., Hu G.W., Han X.B., Gao J., Ma R. (2021). Facile synthesis of magnetic CS-g-SPSS microspheres via electron beam radiation for efficient removal of methylene blue. J. Saudi Chem. Soc..

[101] Zhao Y., Chen T., Gao J., Hu P., Li B., Liang T. (2025). MOFs meet loofah: Controlled preparation of pH-responsive biosorbent via radiation grafting in situ growth for efficient dyes removal. Sep. Purif. Technol..

[102] Du J.F., Dong Z., Yang X., Zhao L. (2021). Facile fabrication of polymeric quaternary ammonium salt hydrogel by radiation for dyes removal from aqueous solution. Radiat. Phys. Chem..

[103] Du J.F., Wu Y.Z., Dong Z., Zhang M.M., Yang X., Xiong H.H., Zhao L. (2022). Single and competitive adsorption between Indigo Carmine and Methyl orange dyes on quaternized kapok fiber adsorbent prepared by radiation technique. Sep. Purif. Technol..

[104] Du J.F., Fan D.C., Dong Z., Yang X., Zhao L. (2022). Fabrication of quaternized sisal fber by electron beam radiation and its adsorption of indigo carmine from aqueous solution. Cellulose.

[105] Xiong H.H., Zeng Z.K., Du J.F., Zhao L. (2023). Fabrication of 3-diethylaminopropylamine modified bamboo fiber prepared by radiation technique and its efficient and recycle adsorption for methyl orange. Desalin. Water Treat..

[106] Du J.F., Zhang M.M., Dong Z., Yang X., Xiong H.H., Zeng Z.K., Chen Z.Y., Zhao L. (2022). Facile fabrication of quaternary ammonium salt modified cotton linter by radiation grafting and its effective removal of methyl orange: Batch and dynamic flow mode studies. Fiber. Polym..

[107] Dong Z., Du J.F., Wang A., Yang X., Zhao L. (2022). Removal of methyl orange and acid fuschin from aqueous solution by guanidinium functionalized cellulose prepared by radiation grafting. Radiat. Phys. Chem..

[108] Du J.F., Xu K., Yang X., Dong Z., Zhao L. (2024). Removal of diclofenac sodium from aqueous solution using different ionic liquids functionalized tragacanth gum hydrogel prepared by radiation technique. Int. J. Biol. Macromol..

[109] Niavarani Z., Breite D., Prager A., Abel B., Schulze A. (2021). Estradiol removal by adsorptive coating of a microfiltration membrane. Membranes.

[110] Liu J., Yuan K.F., Liu J.H. (2021). Preparation of sulfamethazine molecularly imprinted polymer by electron beam irradiation polymerization. IOP Conf. Ser. Earth Environ. Sci..

[111] Peng L.F., Li W.K., Du J.F., Zhang M.M., Zhao L. (2024). Efficient removal of p-nitrophenol from water by imidazolium ionic liquids functionalized cellulose microsphere. Int. J. Biol. Macromol..

[112] Lee H.M., Kim J.H., Kim B.J. (2024). Effects of electron beam irradiation on dimethyl methlyphosphonate adsorption behavior of activated carbon fibers. Energy Convers. Manag..

[113] Chen K., Chen L., Shao H.Y., Li J.Y., Wang H.Y., Mao C.K., Xu G. (2024). Investigation into the characteristics of electron beam-aged microplastics and adsorption behavior of dibutyl phthalate. Chemosphere.

[114] Du J.F., Dong Z., Pi Y.X., Zhao L. (2019). Fabrication of cotton linter-based adsorbents by radiation grafting polymerization for humic acid removal from aqueous solution. Polymers.

[115] Fu L.L., Liu K., Yang J.Y., Zhao Y., Wang Z.J., Tang D.X., Li Y.S., Cheng H.Q. (2024). Adsorption behaviors of ctDNA and biological activities based on polyvinyl alcohol/polyethylene glycol/quaternized chitosan composite hydrogel. Molecules.

[116] Leng F., Li T.T., Li T.F., Xie C., Jiang X.L. (2023). Electron beam irradiation modified carboxymethyl chitin microsphere-based hemostatic materials with strong blood cell adsorption for hemorrhage control. Biomater. Sci..

[117] Chen X.L., Chen G.N., Xie C., Liu G.P., Li N.W., Jin W.Q. (2025). Polyolefin reweaved ultra-micropore membrane for CO_2_ capture. Nat. Commun..

[118] Liu Z.Y., Ma R., Du W.J., Yang G., Chen T. (2021). Radiation-initiated high strength chitosan/lithium sulfonate double network hydrogel/aerogel with porosity and stability for efficient CO_2_ capture. RSC Adv..

[119] Abbasi A., Nasef M.M., Kheawhom S., Majidi R.F., Takeshi M., Lotf E.A., Choong T. (2019). Amine functionalized radiation induced grafted polyolefin nanofibers for CO_2_ adsorption. Radiat. Phys. Chem..

[120] Du M., Zhang Y.Y., Wang Z.Y., Lv M.R., Tang A.Q., Yu Y., Qu X., Chen Z.Q., Wen Q.X., Li A. (2022). Insight into the synthesis and adsorption mechanism of adsorbents for efficient phosphate removal: Exploration from synthesis to modification. Chem. Eng. J..

[121] Awual M.R., Hasan M.N., Hasan M.M., Salman M.S., Sheikh M.C., Kubra K.T., Islam M.S., Marwani H.M., Islam A., Khaleque M.A. (2023). Green and robust adsorption and recovery of Europium(III) with a mechanism using hybrid donor conjugate materials. Sep. Purif. Technol..

[122] Li K.Z., Yuan G.Q., Dong L., Deng G., Duan H.J., Jia Q.L., Zhang H.J., Zhang S.W. (2022). Boehmite aerogel with ultrahigh adsorption capacity for Congo Red removal: Preparation and adsorption mechanism. Sep. Purif. Technol..

[123] Yu Y.M., Mo W.Y., Luukkonen T. (2021). Adsorption behaviour and interaction of organic micropollutants with nano and microplastics-A review. Sci. Total Environ..

[124] Gayathiri M., Pulingam T., Lee K.T., Sudesh K. (2022). Activated carbon from biomass waste precursors: Factors affecting production and adsorption mechanism. Chemosphere.

[125] Geng J., Lin L., Gu F., Chang J.M. (2022). Adsorption of Cr(VI) and dyes by plant leaves: Effect of extraction by ethanol, relationship with element contents and adsorption mechanism. Ind. Crop. Prod..

[126] Wang S.J., Liu X.X., Zhang C.Y., Hu W.J., Liu Y.C., Fu X.Z., Yao J., Sun W. (2025). Adsorption and selective mechanism of Pb^2+^ and Cd^2+^ on the surface of calcined modified attapulgite. Sep. Purif. Technol..

[127] Wang C., Xiong C., He Y.L., Yang C., Li X.T., Zheng J.Z., Wang S.X. (2021). Facile preparation of magnetic Zr-MOF for adsorption of Pb(II) and Cr(VI) from water: Adsorption characteristics and mechanisms. Chem. Eng. J..

[128] Acharya R., Lenka A., Parida K. (2021). Magnetite modified amino group based polymer nanocomposites towards efficient adsorptive detoxification of aqueous Cr (VI): A review. J. Mol. Liq..

[129] Xu G., Xu W.S., Tian S., Zheng W.J., Yang T., Wu Y.X., Xiong Q.Z., Kalkhajeh Y.K., Gao H.J. (2021). Enhanced phosphate removal from wastewater by recyclable fiber supported quaternary ammonium salts: Highlighting the role of surface polarity. Chem. Eng. J..

[130] Zhang L.S., Feng M.H., Zhao D., Li M.M., Qiu S.K., Yuan M.Y., Guo C.B., Han W.J., Zhang K.Q., Wang F. (2023). La-Ca-quaternary amine-modified straw adsorbent for simultaneous removal of nitrate and phosphate from nutrient-polluted water. Sep. Purif. Technol..

[131] Mackay S.E., Malherbe F., Eldridge D.S. (2022). Quaternary amine functionalized chitosan for enhanced adsorption of low concentration phosphate to remediate environmental eutrophication. Colloid Surface A.

[132] Medhat A., Maghrabi H.H., Abdelghany A., Menem N.M., Raynaud P., Moustafa Y.M., Elsayed M.A., Nada A.A. (2021). Efficiently activated carbons from corn cob for methylene blue adsorption. Appl. Surf. Sci. Adv..

[133] Umesh A.S., Puttaiahgowda Y.M., Thottathil S. (2024). Enhanced adsorption: Reviewing the potential of reinforcing polymers and hydrogels with nanomaterials for methylene blue dye removal. Surf. Interfaces.

[134] Harja M., Buema G., Bucur D. (2022). Recent advances in removal of Congo Red dye by adsorption using an industrial waste. Sci. Rep..

[135] Geng R.Y., Wang J., Zhang Z., Dong Q.J., Wu F.F., Chen S.S., Su T., Qi X.L. (2023). Adsorption of antibiotics by polydopamine-modified salecan hydrogel: Performance, kinetics and mechanism studies. Chem. Eng. J..

[136] Wang Z.R., Jang H.M. (2022). Comparative study on characteristics and mechanism of levofloxacin adsorption on swine manure biochar. Biores. Technol..

[137] Ye H., Zhao B.Y., Zhou Y.H., Du J.Y., Huang M.Q. (2021). Recent advances in adsorbents for the removal of phthalate esters from water: Material, modification, and application. Chem. Eng. J..

[138] Ma C.D., Lu T.Y., Shao J.W., Huang J.M., Hu X., Wang L.L. (2022). Biomass derived nitrogen and sulfur co-doped porous carbons for efficient CO_2_ adsorption. Sep. Purif. Technol..

[139] Wang S., Lee Y.R., Won Y., Kim H., Jeong S.E., Hwang B.W., Cho A.R., Kim J.Y., Park Y.C., Nam H. (2022). Development of high-performance adsorbent using KOH-impregnated rice husk-based activated carbon for indoor CO_2_ adsorption. Chem. Eng. J..

[140] Zou Y.H., Huang Y.B., Si D.H., Yin Q., Wu Q.J., Weng Z.X., Cao R. (2021). Porous metal-organic framework liquids for enhanced CO_2_ adsorption and catalytic conversion. Angew. Chem. Int. Ed..

